# Repetitive Head Impact Exposure in College Football Following an NCAA Rule Change to Eliminate Two-A-Day Preseason Practices: A Study from the NCAA-DoD CARE Consortium

**DOI:** 10.1007/s10439-019-02335-9

**Published:** 2019-08-06

**Authors:** Brian D. Stemper, Alok S. Shah, Jaroslaw Harezlak, Steven Rowson, Stefan Duma, Jason P. Mihalik, Larry D. Riggen, Alison Brooks, Kenneth L. Cameron, Christopher C. Giza, Megan N. Houston, Jonathan Jackson, Matthew A. Posner, Gerald McGinty, John DiFiori, Steven P. Broglio, Thomas W. McAllister, Michael McCrea, And the CARE Consortium Investigators, April Marie Hoy, Joseph B. Hazzard, Louise A. Kelly, Justus D. Ortega, Nicholas Port, Margot Putukian, T. Dianne Langford, Ryan Tierney, Christopher C. Giza, Joshua T. Goldman, Holly J. Benjamin, Thomas Buckley, Thomas W. Kaminski, James R. Clugston, Julianne D. Schmidt, Luis A. Feigenbaum, James T. Eckner, Kevin Guskiewicz, Jason P. Mihalik, Jessica Dysart Miles, Scott Anderson, Christina L. Master, Micky Collins, Anthony P. Kontos, Jeffrey J. Bazarian, Sara P. O. Chrisman, Alison Brooks, Jonathan Jackson, Gerald McGinty, Patrick O’Donnell, Kenneth Cameron, Megan N. Houston, Adam Susmarski, Stefan Duma, Steve Rowson, Christopher Todd Bullers, Christopher M. Miles, Brian H. Dykhuizen, Laura Lintner

**Affiliations:** 1 Joint Department of Biomedical Engineering, Marquette University and Medical College of Wisconsin, Milwaukee, WI USA; 2 Department of Neurosurgery, Medical College of Wisconsin, Milwaukee, WI USA; 3 Neuroscience Research, Clement J. Zablocki Veterans Affairs Medical Center, Milwaukee, WI USA; 4 Department of Epidemiology and Biostatistics, Indiana University School of Public Health, Bloomington, IN USA; 5 Department of Biomedical Engineering and Mechanics, Virginia Tech, Blacksburg, VA USA; 6 Matthew Gfeller Sport-Related Traumatic Brain Injury Center, University of North Carolina at Chapel Hill, Chapel Hill, NC USA; 7 Department of Orthopedics, School of Medicine and Public Health, University of Wisconsin, Madision, WI USA; 8 John A. Feagin Jr. Sports Medicine Fellowship, Keller Army Hospital, United States Military Academy, West Point, NY USA; 9 Department of Sports Medicine, United States Air Force Academy, Colorado Springs, CO USA; 10 Michigan Concussion Center, University of Michigan, Ann Arbor, MI USA; 11 Department of Psychiatry, Indiana School of Medicine, Indianapolis, IN USA; 12 Departments of Neurosurgery and Pediatrics, UCLA Steve Tisch BrainSPORT Program, David Geffen School of Medicine, University of California, Los Angeles, Los Angeles, CA USA; 13 Hospital for Special Surgery, New York, NY USA

**Keywords:** Sport-related concussion, Injury biomechanics, Traumatic brain injury, Acceleration

## Abstract

Repetitive head impact exposure sustained by athletes of contact sports has been hypothesized to be a mechanism for concussion and a possible explanation for the high degree of variability in sport-related concussion biomechanics. In an attempt to limit repetitive head impact exposure during the football preseason, the NCAA eliminated two-a-day practices in 2017, while maintaining the total number of team practice sessions. The objective of this study was to quantify head impact exposure during the preseason and regular season in Division I college football athletes to determine whether the 2017 NCAA ruling decreased head impact exposure. 342 unique athletes from five NCAA Division I Football Bowl Subdivision (FBS) programs were consented and enrolled. Head impacts were recorded using the Head Impact Telemetry (HIT) System during the entire fall preseasons and regular seasons in 2016 and 2017. Despite the elimination of two-a-day practices, the number of preseason contact days increased in 2017, with an increase in average hourly impact exposure (i.e., contact intensity), resulting in a significant increase in total head impact burden (+ 26%) for the 2017 preseason. This finding would indicate that the 2017 NCAA ruling was not effective at reducing the head impact burden during the football preseason. Additionally, athletes sustained a significantly higher number of recorded head impacts per week (+ 40%) during the preseason than the regular season, implicating the preseason as a time of elevated repetitive head impact burden. With increased recognition of a possible association between repetitive head impact exposure and concussion, increased preseason exposure may predispose certain athletes to a higher risk of concussion during the preseason and regular season. Accordingly, efforts at reducing concussion incidence in contact sports should include a reduction in overall head impact exposure.

## Introduction

Understanding the deleterious effects of repetitive subconcussive head impact exposure through routine participation in contact sports has come to the forefront of sport-related concussive injury prevention efforts. Recent reports indicated that elevated levels of repetitive head impact exposure in contact sports may be a second biomechanical mechanism for concussion^34^,^35^ or a contributing factor to cognitive and MRI changes in non-concussed athletes.^3^,^15^,^28^,^33^ This second concussion mechanism is separate from the traditionally-accepted concussion mechanism that involves a single head impact resulting in high rate head rotational acceleration.^17^,^29^ Supporting evidence for this second concussive mechanism was provided in observational studies of non-concussed athletes. For example, in a study of National Collegiate Athletic Association (NCAA) Division I athletes that did not sustain concussion, McAllister *et al.* identified significant correlations between head impact exposure metrics and post-season measures of white matter diffusivity in several brain regions.^26^ These findings imply the presence of accumulating structural and physiological changes in the brain that are correlated to repetitive head impact exposure and occur without the diagnosis of concussion. Similarly, other studies identified post-season cognitive changes in non-concussed athletes with higher levels of head impact exposure throughout the season.^24^,^36^ This points to a possible correlation between increasing levels of repetitive head impact exposure and the development of cognitive changes during a football season.

In addition to the identification of cognitive and brain structural changes in non-concussed athletes, recent studies hypothesized that accumulation of damage from repetitive head impacts may eventually contribute to the onset of diagnosed concussion. For example, Beckwith *et al.* reported that high school and college football athletes sustained a higher frequency of impacts and more severe impacts on days of diagnosed concussion than on days without concussion.^5^ Similarly, a recent study reported that some concussed college football athletes sustained more severe repetitive head impact exposure compared to their position- and team-matched controls leading up to concussion.^34^ Many of these “high exposure” athletes sustained concussion after relatively unremarkable concussive head impacts. For example, 40% of “high exposure” athletes in that study had concussive impacts less than 65 g and 22% had concussive impacts less than 50 g. The study recorded over 6500 head impacts greater than 65 g and over 14,000 head impacts greater than 50 g that did not result in concussion. Varying levels of repetitive head impact and concussion history is thought to be a possible contributor to individual-specific concussive tolerance^31^ that may explain the significant variability reported for concussive biomechanics in contact sports.^6^,^16^,^25^ These findings highlight a possible biomechanical mechanism involving progressive damage in the brain resulting from repetitive subconcussive head impacts. Accordingly, more frequent or more severe head impacts can decrease the tolerance for concussion, making the athlete more susceptible to injury from lower magnitude head impacts.

Recently, field studies of college football athletes indicated that concussive injuries are reported with higher frequency during specific periods of the season and during specific activities. For example, Krill *et al.* indicated that the highest rate for musculoskeletal and concussive injuries in college football athletes was during the preseason, prior to the initiation of regular season games.^23^ A majority of injuries in that study occurred under a contact mechanism. Focusing specifically on concussion, a recent study demonstrated that over half of the 50 concussions occurred in the month of August, prior to the initiation of games.^34^ The August college football preseason is likely a time of elevated head impact exposure due to the frequency of contact activities associated with two-a-day practices and a higher number of team activities per week compared to the regular season. Accordingly, elevated levels of repetitive head impact exposure during the college football preseason may be contributing to higher rates of concussion reported during that time period in the studies highlighted above.

Based on the types of information highlighted above, concussion prevention efforts by college football governing bodies have begun to focus on limiting athlete head impact burden during practice activities. For example, Ivy League coaches voted to eliminate full contact hitting from practices during the 2016 regular season.^7^ In 2017, the NCAA acted to eliminate two-a-day practices during the preseason for all Division I Football Bowl Subdivision (FBS) programs.^37^ Although not explicitly stated by the NCAA, this effort would appear to be targeted at reducing head impact density (i.e., number of head impacts over time) and total head impact burden for NCAA Division I college football athletes during the preseason. The effect of the 2017 NCAA ruling on head impact burden and concussion incidence during the Division I college football preseason has not yet been reported. Accordingly, the objectives of the current analysis were to profile subconcussive head impact exposure during the 2016 and 2017 college football preseasons and to compare preseason to regular season head impact exposure. Based on the information highlighted above, the hypotheses were that preseason head impact density would exceed that of the regular season and that total head impact burden would decrease for athletes during the 2017 preseason.

## Methods

This study was conducted to profile head impact exposure in non-concussed athletes across two football seasons in response to a 2017 NCAA preseason practice schedule change affecting Division I FBS programs. Although the ruling was focused on the 2017 preseason practice schedule, data are presented for the fall preseasons and regular seasons for 2016 and 2017 to outline the effect of the ruling on preseason exposure and highlight differences in head impact burden between the preseason and regular season. Data included in this investigation are a subset of the NCAA-DoD Grand Alliance Concussion Assessment, Research and Education (CARE) Consortium. Methods were described elsewhere^10^ and the study protocol was approved by the Institutional Review Board (IRB) at the Medical College of Wisconsin (MCW), with local sites utilizing a reliance agreement with the MCW IRB. The study also obtained approval from the US Army Human Research Protection Office (HRPO).

### Details of the 2017 NCAA Rule Change

The 2017 NCAA rule change focused on the elimination of 2-a-day practices. During the 2016 preseason, multiple on-field practices per day (e.g., two-a-days or three-a-days) were allowed, but not on consecutive days. Multiple on-field practices per day were eliminated in 2017. Both years had a 3-hour limit for on-field practice activities during days in which there was only one practice. However, in 2016, a 5-hour limit for on-field practice time was imposed on days during which there were multiple on-field practices. Three hours of recovery time was required between two on-field practices in the same day. Both years limited the total number of preseason on-field practice sessions to 29, with two-a-day practices counting as two on-field sessions during the 2016 preseason. The date of the first allowable practice activity was limited to 40 units prior to the first intercollegiate game during both years. There were no changes to the regular season game or practice schedule in 2017.

### Head Impact Data Collection

Varsity college football athletes from five NCAA Division I FBS programs were consented during the 2016 and 2017 seasons. Those programs included the United States Air Force Academy, The United States Military Academy at West Point, the University of North Carolina at Chapel Hill (UNC), the University of Wisconsin (UW), and Virginia Tech (VT). Each athlete was equipped with the helmet-based Head Impact Telemetry (HIT) System in Riddell Speed or Speed Flex helmets (Riddell SRS, Riddell, Rosemont, IL, USA), which measures helmet linear accelerations using six uniaxial accelerometers inside the football helmet. Head impact data are stored onboard the helmet instrumentation until the helmet comes within range of a sideline computer, at which point the data are wirelessly transmitted to the sideline computer. Data acquisition triggered any time a single accelerometer exceeded a 9.6-g threshold. The HIT System computes peak resultant and component linear and rotational accelerations. Any impacts with peak resultant linear acceleration below 10 g were not included in this analysis as they can be associated with non-impact dynamic movements in the athlete.^12^,^22^,^31^

Data collection was managed by HIT System operators at each Institution. The operators placed battery-charged sensors in each helmet prior to every contact practice, scrimmage and game. The sensors turned on when the helmet was placed on the athlete’s head. Operators defined a “session” prior to every contact practice, scrimmage and game, which initiated data collection by the sensors and wireless transfer of data from the helmet instrumentation to the sideline recording unit. Operators charged the sensor batteries once per week and offloaded any head impacts that were not wirelessly transmitted from the helmet instrumentation to the sideline computer during practices and games. Data were then uploaded and stored in the Riddell cloud. The Investigative team was provided access to the HIT System data in the cloud or by direct transfer from the local site *via* a secure file transfer protocol (ftp) server. Each athlete was provided a unique study specific number to prevent disclosure of personal identifiable information.

Head impact data were collected using the HIT System for all practice, scrimmage, and game activities involving contact, initiating for August pre-season practices and continuing through the end of the regular season for each team. Analyzed data do not include conference championship or bowl games. Data were synced daily to the Riddell cloud from each Institution and accessed by the Investigative Team for quality control and analysis. Quality control consisted of confirming that HIT data were consistent with practice and game dates/times outlined on activity logs maintained by local study coordinators, checking HIT System output files to ensure that all athlete and impact information values were included, filtering out any impacts that exceeded 200 g and 10,000 rad/s^2^ which was a decision made by the CARE Head Impact Measurement Core, and cross-checking enrollment records to ensure that we did not receive any HIT System data for athletes not enrolled in this study.

### Team-Based Changes in Preseason Schedule

Several metrics were quantified for the five participating teams during the 2016 and 2017 football preseasons to assess changes in preseason practice schedules associated with the 2017 NCAA rule change. The number of contact practice days, the number of contact practice sessions, the duration of contact practice sessions, and the number of two-a-day practices were quantified for each participating team. These metrics were determined by analyzing the timing of head impacts for each day across all athletes on a given team. Contact practice days were counted as the total number of preseason days for which head impacts were recorded for each team. Contact practice sessions were assessed as distinct periods of head impact activity on any given preseason day. Two-a-day contact practices were identified as two distinct periods of head impacts on a single preseason day, separated by an extended period of time (typically 3 + hours). Additional two-a-day on-field practices may have also occurred, with one contact and one non-contact session, but non-contact sessions were not able to be tracked in the current analysis that was focused on periods of head impact activity. Contact practice duration was quantified as the duration of time between the first and last head impact for a given team on each preseason day. Finally, the start date for preseason contact practices was noted for each team and compared between the 2016 and 2017 seasons.

### Analysis of Athlete Exposure

The number of recorded head impacts per week was calculated for each athlete over the course of the entire preseason and regular season. For the purpose of this study, each week initiated on Monday and concluded on the following Sunday. Preseason weeks were represented as negative numbers counting backward from the start of the regular season, with the last preseason week as − 1 and the first preseason week as − 4 or − 5. Regular season weeks initiated on the Monday leading up to the first regular season game (Week 1) and were numbered consecutively through the end of the regular season (Week 13). The number of impacts per week were averaged and compared across seasons and playing positions. Player position groups included defensive linemen (DL), linebackers (LB), defensive backs (DB; cornerbacks and safeties), offensive linemen (OL), running backs (RB; running backs and full backs), tight ends (TE), wide receivers (WR), and quarterbacks (QB). Kickers and punters were not included in the analysis.

In addition to the number of recorded head impacts per week, a number of other metrics were quantified to describe athlete exposure during the fall preseason and regular season. The number of head impacts per day was calculated as the total number of recorded head impacts for each date of participation for each athlete. The total number of head impacts was computed for the preseason and regular season for each athlete. The number of contact days was also computed for each athlete as the number of days in which the athlete sustained at least one head impact. Each of these metrics was compared between the preseason and regular season, between primary playing positions, and between study years (2016 vs. 2017).

In addition to quantifying head impact burden on a daily, weekly, and seasonal basis, contact intensity was quantified as the number of head impacts per hour. This metric captured the intensity of activities during contact practices and competitions, and was designed to normalize the number of head impacts over the specific period of time that they were sustained. The total number of head impacts recorded during each athlete session was divided by the time duration between the first and last recorded head impact for that session. Athlete sessions with only one recorded head impact were not included in this analysis. Athlete sessions lasting less than 15 min were also removed from the analysis to prevent artificially high values for contact intensity. Contact intensity was compared between preseason practice sessions, regular season practice sessions, and games.

### Statistical Analysis

The metrics described above were analyzed for statistically significant differences (*p* < 0.05) between playing positions, years, and period of the season (preseason vs. regular season). The distribution of each of these metrics was assessed for normality using a Shapiro–Wilk test, with the null hypothesis of normality. Metrics with non-normal distributions were assessed for significant group-wise differences using a Kruskal–Wallis equality-of-populations rank test.

## Results

All five NCAA Division I FBS programs participated in the study during the 2016 and 2017 seasons and had the same head coaches during both seasons. A total of three hundred forty-two (342) unique athletes were consented, enrolled, and included in the present analysis for a total of 459 athlete-seasons. Two hundred twenty-five (225) athletes from five NCAA Division I football programs participated in contact activities over four preseason and thirteen regular season weeks during 2016, for a total of 10,861 athlete-days. Two hundred thirty-four (234) athletes from the same five NCAA Division I football programs participated in contact activities over five preseason and thirteen regular season weeks during 2017, for a total of 10,647 athlete-days. The distribution of athletes by position group is provided in Table 1. A total of 141,558 head impacts were recorded during the 2016 fall season and 135,140 head impacts were recorded during the 2017 fall season.

**Table 1:** Enrollment by primary position group during the preseason and regular season for both years of this study.

	2016		2017	
	Preseason	Regular season	Preseason	Regular season
Defensive backs (DB)	38	42	34	35
Defensive line (DL)	36	35	38	36
Linebackers (LB)	30	31	34	33
Offensive line (OL)	41	45	48	49
Quarterbacks	7	8	9	10
Running backs (RB)	18	19	17	17
Tight ends (TE)	11	11	14	13
Wide receivers (WR)	16	22	28	27
Total	197	213	222	220

### Team-Based Preseason Schedule Changes

The five enrolled teams participated in between 0 and 5 two-a-day preseason practice dates in 2016 (average: 2.6 ± 2.0). All teams had zero two-a-day practice dates in 2017, according to the updated NCAA rule. The average number of preseason contact practice days across the five teams increased (+ 15.4%; *p* = 0.076) from 18.2 ± 1.8 days in 2016 to 21.0 ± 3.5 days in 2017. All five teams had a greater number of preseason practice days in 2017. However, when accounting for two-a-day practices in 2016, the number of preseason contact practice sessions remained essentially the same (*p* = 0.595) between 2016 (20.8 ± 2.9) and 2017 (21.0 ± 3.5). Similarly, average preseason practice duration was consistent (*p* = 0.465) between 2016 (2.18 ± 0.27 h) and 2017 (2.17 ± 0.32 h). Relative to the start of Week 1 of the regular season (i.e., the Monday prior to the first game), teams had their first preseason contact practice in 2017 between 0 and 9 days earlier than 2016. Two teams initiated practice 0 and 1 day earlier and the other three teams initiated practice 5, 6, and 9 days earlier.

### Athlete Total Head Impact Burden: Preseason and Regular Season

Comparison of the total number of recorded head impacts between the two years of this study revealed interesting trends. Across all positions, athletes saw an increased number of recorded head impacts during the 2017 preseason (*p* < 0.05) and a decrease in the number of recorded head impacts during the 2017 regular season (*p* < 0.05). Overall, athletes sustained an average of 26% more head impacts during the preseason in 2017, and 20% fewer total head impacts during the 2017 regular season (Table 2). All playing position groups except receivers saw an increase in the total number of recorded preseason head impacts in 2017 with increases between 13% (TE) and 49% (RB). Conversely, all position groups saw a decrease in total regular season head impact exposure of between 14% (DB, DL) and 54% (WR).

**Table 2:** Head impact exposure data for the preseason and regular season of the 2016 and 2017 seasons from five NCAA Division I FBS programs.

	Preseason			Regular Season		
	2016	2017	% Change	2016	2017	% Change
Impacts per day						
Overall	12.6 ± 8.6	11.5 ± 7.8	− 9	12.4 ± 7.9	11.3 ± 7.6	− 8
Defensive back	7.3 ± 4.2	8.2 ± 4.3	+ 12	7.6 ± 4.0	7.4 ± 4.0	− 3
Defensive line	18.7 ± 10.1	16.4 ± 7.1	− 12	16.9 ± 7.6	17.2 ± 7.4	+ 2
Linebacker	11.3 ± 5.1	10.5 ± 6.2	− 7	12.7 ± 7.4	10.7 ± 6.1	− 16
Offensive line	19.4 ± 7.9	16.9 ± 9.0	− 13	18.1 ± 9.0	15.9 ± 8.3	− 12
Quarterback	3.2 ± 1.6	3.4 ± 1.2	+ 6	7.3 ± 3.9	5.7 ± 2.8	− 22
Running back	7.7 ± 4.6	8.9 ± 3.7	+ 16	10.9 ± 4.9	9.2 ± 6.0	− 16
Tight end	12.6 ± 5.6	11.9 ± 6.1	− 6	11.1 ± 3.9	10.5 ± 4.8	− 5
Wide receiver	6.4 ± 3.9	5.0 ± 4.2	− 22*	5.9 ± 3.3	4.9 ± 3.3	− 20
Total contact days						
Overall	11.5 ± 5.3	14.8 ± 5.6	+ 29***	40.4 ± 16.5	33.5 ± 19.7	− 17***
Defensive back	11.7 ± 5.5	14.8 ± 5.6	+ 26*	38.3 ± 18.3	33.4 ± 20.4	− 13
Defensive line	10.5 ± 5.2	15.0 ± 5.1	+ 43***	45.4 ± 11.4	38.5 ± 16.8	− 15**
Linebacker	10.1 ± 6.0	15.7 ± 5.9	+ 55**	39.4 ± 19.0	32.8 ± 21.5	− 17
Offensive line	12.2 ± 4.8	15.2 ± 6.0	+ 25**	40.6 ± 17.4	38.4 ± 20.6	− 5
Quarterback	11.1 ± 2.9	13.6 ± 4.7	+ 23*	30.6 ± 13.5	25.2 ± 10.4	− 18
Running back	11.2 ± 6.0	15.5 ± 5.0	+ 38**	39.3 ± 15.6	31.2 ± 22.9	− 21
Tight end	14.6 ± 2.7	17.1 ± 4.6	+ 17*	48.4 ± 10.6	35.3 ± 19.4	− 27
Wide receiver	12.1 ± 5.9	11.3 ± 5.1	− 7	37.8 ± 16.4	22.6 ± 15.4	− 40**
Total impacts						
Overall	152 ± 126	191 ± 162	+ 26**	524 ± 403	421 ± 417	− 20***
Defensive back	99 ± 86	133 ± 97	+ 34*	303 ± 205	262 ± 227	− 14
Defensive line	201 ± 144	263 ± 158	+ 31*	790 ± 405	680 ± 413	− 14
Linebacker	125 ± 102	185 ± 140	+ 48*	489 ± 328	384 ± 347	− 21
Offensive line	238 ± 144	288 ± 207	+ 21	753 ± 501	637 ± 545	− 15
Quarterback	36 ± 20	49 ± 26	+ 36	200 ± 142	144 ± 86	− 28
Running back	99 ± 71	148 ± 91	+ 49*	460 ± 299	313 ± 306	− 32
Tight end	189 ± 93	214 ± 135	+ 13	552 ± 295	397 ± 324	− 28**
Wide receiver	87 ± 70	66 ± 69	− 24	261 ± 212	120 ± 127	− 54**

Data are presented as the average number of impacts per day, the total number of contact days, and the total number of recorded head impacts for the preseason and regular season, averaged across the entire sample and also presented by position. Statistical results are presented for year-to-year differences as *p < 0.1; **p < 0.05; ***p < 0.001

Averaging across all enrolled athletes, 2016 athletes sustained 25.2 ± 24.3% (mean ± standard deviation) of their total season-long head impact burden during the preseason. In contrast, 2017 athletes sustained 37.3 ± 24.7% of their total season-long head impact burden during the preseason. That accounts for an increase of 48% (*p* < 0.0001) in the preseason contribution to the total season-long head impact burden from 2016 to 2017. Analyzed separately, each position group also demonstrated an increase in the percentage of total season-long recorded head impacts during the preseason from 2016 to 2017. Preseason burden increased between 30% and 78% from 2016 to 2017 for all position groups except quarterbacks and wide receivers, who demonstrated 120% and 115% increases in preseason burden. These increases from 2016 to 2017 were statistically significant (*p* < 0.05) for all positions except defensive backs and linebackers.

### Athlete Weekly Head Impact Exposure

**Figure 1:**
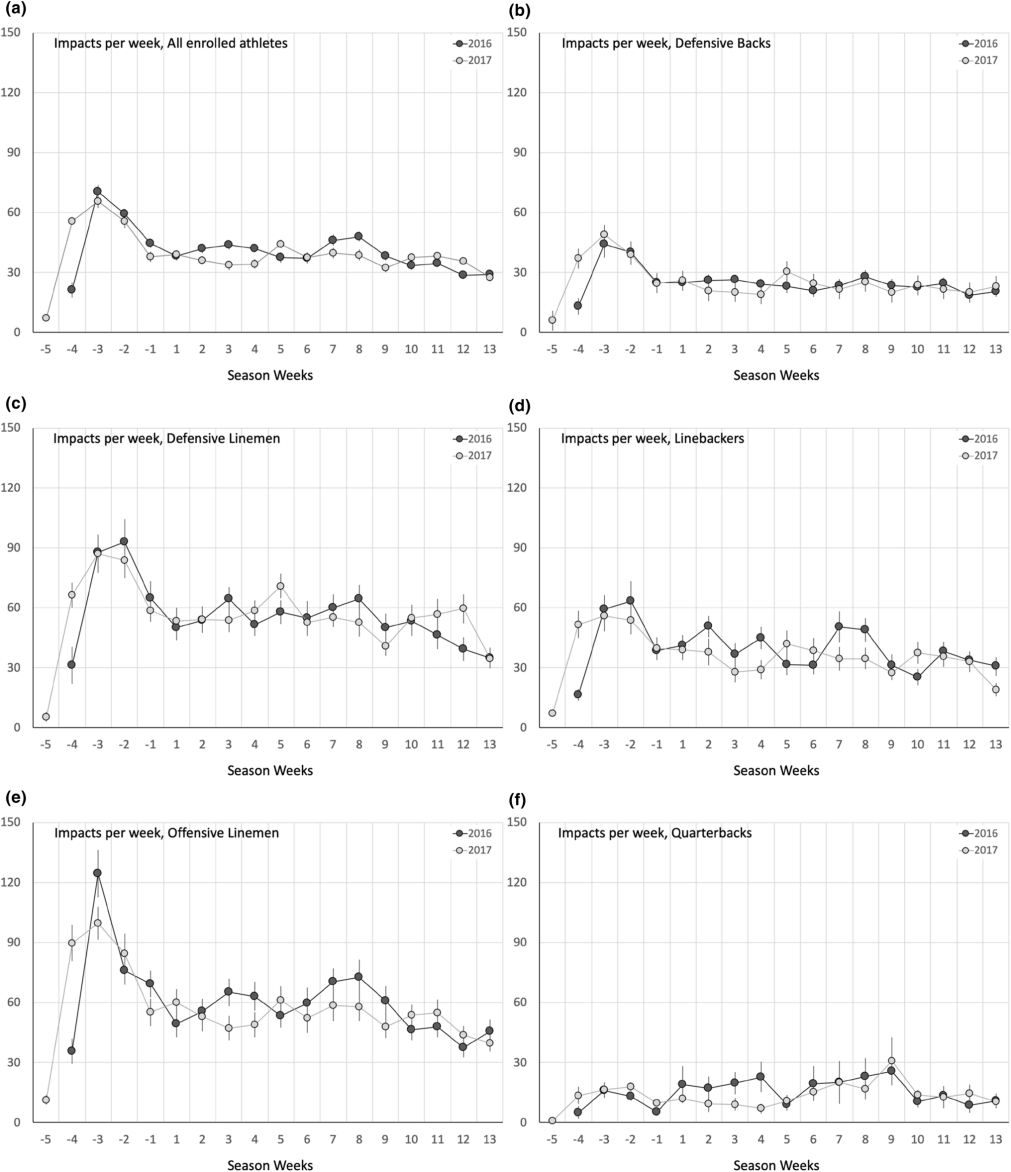

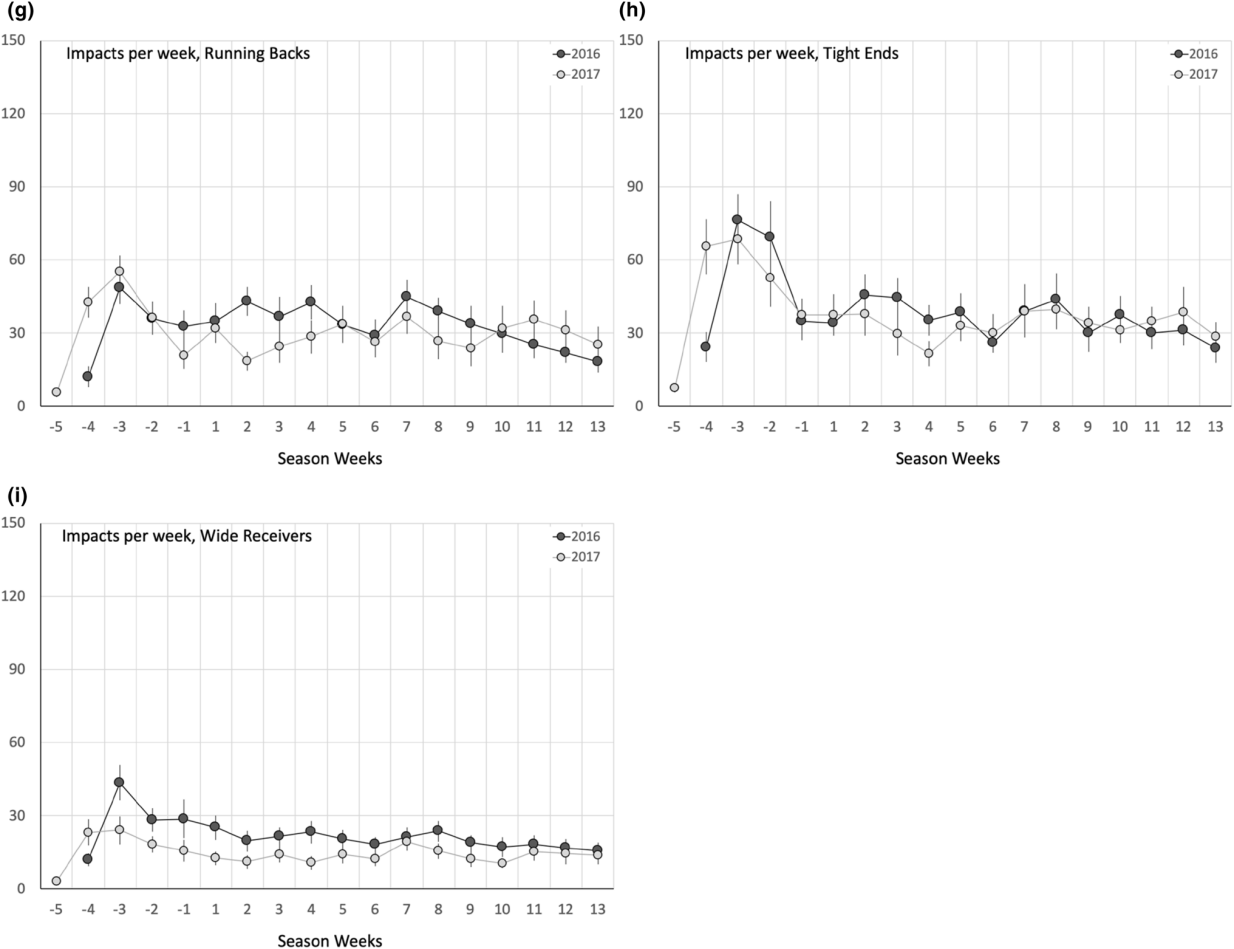
Average number or recorded head impacts per week for each of the eight position groups analyzed in this study. Dark lines/dots represent data from 2016 and light lines/dots represent data from 2017. Position groups include (b) defensive backs, (c) defensive linemen, (d) linebackers, (e) offensive linemen, (f) quarterbacks, (g) running backs, (h) tight ends, and (i) wide receivers. Overall position-independent data (a) are also presented.

The profile of weekly head impact exposure (i.e., number of recorded head impacts per week) across all non-concussed athletes generally decreased from the early preseason through the end of the regular season (Fig. 1). This trend was evident for both the 2016 and 2017 seasons. However, the change in head impact exposure from week to week was more dramatic during the preseason than the regular season. Athletes had remarkably higher head impact exposure earlier in the preseason (e.g., weeks − 4 to − 2) that decreased toward the start of the regular season. Regular season head impact exposure remained relatively consistent from week to week with only a slight decreasing trend over the course of the regular season. Overall, average number of recorded head impacts per week during the preseason (51.5 ± 47.1 impacts/week; mean ± standard deviation) was significantly greater (*p* < 0.0001) than the average number of recorded head impacts per week during the regular season (36.7 ± 32.8 impacts/week). The preseason/regular season difference in weekly head impact exposure was particularly evident when focusing on the early preseason. For example, athletes sustained an average of as many as 70.4 head impacts per week earlier in the preseason (2016 Week − 3), which was 92% greater than the average regular season weekly head impact exposure.

The number of recorded head impacts per week also significantly varied (*p* < 0.05) by athlete position group (Fig. 1). Offensive linemen (57.8 ± 45.4 impacts per week; mean ± standard deviation) and defensive linemen (56.5 ± 39.8 impacts per week) sustained the highest number of head impacts per week throughout the 2016 and 2017 seasons. Tight ends (37.5 ± 29.3 impacts per week) and linebackers (37.3 ± 29.7 impacts per week) sustained the third and fourth highest number of weekly head impacts. Quarterbacks (13.7 ± 14.1 impacts per week) sustained by far the fewest number of recorded head impacts per week. The number of recorded head impacts per week during the preseason was significantly greater than the regular season (*p* < 0.0001) for each position group except quarterbacks and running backs. The position groups with significantly increased preseason exposure demonstrated average position-based increases in weekly exposure between 26 and 62% (average: 44%) for the preseason compared to the regular season.

The number of recorded head impacts per week was generally consistent between the 2016 and 2017 seasons. Preseason weekly head impact exposure across all playing positions decreased by 4.9% (*p* = 0.1187) in 2017 compared to 2016. Regular season weekly head impact exposure across all playing positions decreased by 4.7% (*p* = 0.0026) in 2017 compared to 2016. Wide receivers demonstrated the largest change in weekly head impact exposure from 2016 to 2017, with a 34.9% decrease (*p* < 0.05) during the preseason and a 33.2% decrease (*p* < 0.05) during the regular season. Linebackers (− 12%), offensive line (− 8.9%), and quarterbacks (− 14.3%) all demonstrated significant decreases (*p* < 0.05) in weekly head impact exposure during the regular season in 2017.

### Athlete Number of Contact Days

Differences in total head impact exposure from 2016 to 2017 were consistent with change in the number of days of contact activities between those two years. Across all playing positions, athletes were exposed to 29% more days of preseason contact activities in 2017, while the number of regular season contact days decreased by 17%. The increase in preseason contact days was primarily attributable to the earlier start of contact practices in 2017 (July 29) compared to 2016 (August 5), which permitted a greater number of contact practice activities prior to the first game of the season. Conversely, athletes saw a 17% decrease in the number of contact days during the regular season. Focusing on differences by athlete position, a majority of positions saw an increase of 17% (TE) to 55% (LB) in preseason contact days from 2016 to 2017. Wide receivers saw a 7% decrease in contact days during the 2017 preseason. Similarly, all eight position groups saw a decrease in the number of contact days during the regular season in 2017, with between 5% (OL) and 40% (WR) fewer contact days.

### Athlete Daily Head Impact Exposure

The number of head impacts per day was consistent between the preseason and regular season (Table 2). Across all playing positions, athletes sustained 12.0 ± 8.2 head impacts per day during the preseason and 11.8 ± 7.7 head impacts per day during the regular season. On a season-to-season basis, the number of head impacts per day marginally decreased by 9% (p > 0.05) for the preseason and 8% (p > 0.05) for the regular season in 2017. None of the position groups demonstrated a statistically significant change in daily head impact exposure during the preseason or regular season between 2016 and 2017.

This analysis also identified significant differences in head impact exposure between practices and games. In both 2016 and 2017, athletes sustained a significantly higher number of head impacts (*p* < 0.0001) during regular season games (19.7 ± 18.0) than regular season practices (10.4 ± 10.9). Year-to-year differences were also evident when separating regular season activities between practices and games. Across all playing positions, the number of head impacts per session significantly increased (*p* < 0.05) during regular season practices in 2017 (10.6 ± 11.0) compared to 2016 (10.1 ± 10.7) and significantly decreased during regular season games in 2017 (18.9 ± 17.2) compared to 2016 (20.4 ± 18.6). However, across all playing positions, the number of head impacts per session was not significantly different between individual practice sessions (12.5 ± 11.5) and scrimmages (12.6 ± 10.9) during the preseason. Focusing on year-to-year preseason differences, across all playing positions, the number of head impacts per practice session increased (*p* < 0.0001) during preseason practices in 2017 (13.1 ± 12.1) compared to 2016 (11.8 ± 10.6). The number of recorded head impacts per preseason scrimmage only slightly decreased (*p* < 0.1) in 2017 (12.0 ± 10.3) compared to 2016 (13.6 ± 11.8).

### Athlete Contact Intensity

Contact intensity was assessed as the number of head impacts recorded for each athlete session, divided by the duration from the first to the last impact for that session. Contact intensity was computed separately for both practice sessions of each two-a-day practice. Average preseason contact intensity per athlete session increased by 10.8% from 2016 (9.3 ± 6.3 impacts/hour) to 2017 (10.3 ± 6.9 impacts per hour). Regular season contact intensity also increased in 2017, but to a lesser degree. Average regular season contact intensity was 9.5 ± 7.1 impacts per hour in 2016 and 10.0 ± 7.7 impacts per hour in 2017.

### Concussion Occurrence

Athletes enrolled in this study sustained a total of 37 concussions during the 2016 and 2017 preseason and regular seasons. Seventeen concussions occurred in 2016, with 8/17 (47%) occurring in the month of August. Twenty concussions occurred in 2017, with 10/20 (50%) occurring in the month of August. This represents a 17.6% increase in the number of concussions for enrolled athletes from 2016 to 2017. The rate of concussions was also 19.7% greater in 2017 (1.88 per 1000 athlete days) compared to 2016 (1.57 per 1000 athlete days). However, given the limited number of total concussions in this current analysis, this increase should not be interpreted as a statistically significant increase. Likewise, the increase in concussions in 2017 could be attributed to factors other than the increased preseason head impact exposure during that preseason. Nonetheless, the data are presented here for the purpose of completeness as concussion occurrence was tracked in the current study and may have been influenced by greater preseason head impact exposure.

## Discussion

This study characterized repetitive head impact exposure in a large sample of NCAA Division I college football athletes, with a focus on identifying differences in athlete head impact burden during the preseason and regular season and between the years 2016 and 2017. Those years were chosen for this analysis due to the NCAA rule change in 2017 that eliminated two-a-day practices. Focusing on preseason head impact exposure, this investigation revealed a significant increase in the total preseason head impact burden in 2017. This finding was somewhat surprising given the 2017 NCAA rule change that was presumably intended to decrease preseason head impact exposure. The 2017 increase in preseason head impact exposure occurred without significant increases in athlete weekly or daily exposure, or the number of team practice sessions. However, the number of recorded head impacts per session significantly increased for 2017 athletes which was the direct result of increased preseason practice intensity (head impacts per hour), as team practice durations did not increase in 2017. Although somewhat limited in scope, increased preseason head impact exposure for these five NCAA Division 1 programs may be representative of how other programs across the NCAA adjusted preseason schedules to account for this rule change. Greater preseason head impact exposure may have predisposed some athletes to a greater risk of concussion in 2017 during a time of the season already associated with greater injury risk.

Another major finding from this investigation was the quantification of increased head impact exposure during the fall preseason compared to the regular season. On a weekly basis, athletes sustained 40% more head impacts during the preseason than the regular season. This trend was consistent across both years of the study, although the percentage of season-long head impacts occurring in the preseason increased from 25% in 2016 to 37% in 2017. These findings have the potential for major significance, given the recent identification of a correlation between incident concussion and high levels of head impact exposure,^34^,^35^ which may implicate an elevated number of repetitive head impacts as a mechanism of concussion for some athletes. Accordingly, a 26% increase in number of head impacts sustained during preseason training camp in 2017 may have predisposed some athletes to a greater risk of concussion both during the preseason and regular season of that year. In fact, athletes enrolled in this study sustained approximately 18% more concussions during the preseason and regular season in 2017, with greater than 15% increase in concussion rate per athlete exposure. However, the total number of concussions (n = 37) in this two-year analysis is likely too small to make definitive conclusions. Concussion incidence for all football athletes across the entire NCAA Division I FBS were not available at the time of this analysis.

This increase in exposure and concussion rate from 2016 to 2017 comes at a time of increased recognition of the possible effects of repetitive head impact exposure. Field studies of amateur football athletes have reported significant correlations between the number and severity of repetitive head impacts and clinical or advanced MRI changes in non-concussed athletes.^1^,^3^,^8^,^24^,^26^,^28^,^36^ A fewer number of studies have also begun to focus on the possible link between repetitive head impact exposure and concussive injury.^5^,^11^,^18^,^34^,^35^ Amateur football governing bodies have reacted with policy changes intended to decrease head impact exposure during practice activities such as elimination of two-a-day practices by the NCAA and incorporation of alternatives for tackling drills in practice.^7^ This study was motivated by the NCAA rule change that eliminated two-a-day practices in 2017, which was presumably intended to reduce the frequency and burden of head impact exposure. Results of this study demonstrated a relatively modest effect of that ruling in reducing weekly head impact burden during the preseason (− 2.2%) and the unanticipated effect of increasing the total head impact burden by over 25% across the entire preseason. This was likely attributable to increased contact intensity (impacts per hour) during the 2017 preseason, resulting in a greater number of preseason head impacts over the same number of preseason team contact practice sessions.

Future efforts at reducing head impact burden are likely better targeted at reducing the total head impact burden for the preseason instead of daily head impact exposure, acknowledging that the effect of this ruling on concussion rate across the entire NCAA has not yet been ascertained. Reducing the intensity of contact practices may be the most effective method to reduce total head impact burden, given the association between daily contact intensity and total preseason head impact burden identified here. This finding is consistent with the recent recommendations by Kelley *et al.*, that concluded reducing the speed of players engaged in contact, correcting tackling technique, and progressing to contact as more effective methods to reduce head impact exposure in youth football.^20^ Based in part on data presented here, the NCAA did act again in 2018 to reduce total head impact burden by reducing the total number of on-field preseason practices from 29 in 2016 and 2017 to 25 in 2018. The reduced number of practices likely produced a lower head impact burden in 2018, although data for that season have not yet been presented.

This study focused on quantifying weekly and total head impact burden for college football athletes across two preseasons and regular seasons, focusing on the number of recorded head impacts. These metrics are presented here as a possible correlate for the risk of concussion. Other metrics to quantify head impact burden were reported previously. Risk-weighted exposure (RWE) was proposed by Urban *et al.*^38^ as the cumulative concussive risk for all head impacts sustained over the period of interest. RWE was shown to be elevated in a high percentage of concussed athletes, which may demonstrate its utility as a correlate for concussion risk.^34^ That study reported a stronger correlation of elevated RWE in concussed athletes with the number of recorded head impacts than the severity, as the profile of head impact magnitudes over the period of interest was relatively consistent between concussed and non-concussed athletes. This was particularly true for athletes that did not sustain a significant head impact at the time of their concussion. Therefore, the current focus on the number of recorded head impacts during the preseason and regular season is justified. However, other metrics to quantify cumulative head impact burden have been proposed and should be considered in future assessments of head impact burden. Some of those metrics have focused on life-long exposure, instead of daily, weekly, or season-long exposure.^21^,^28^ Those metrics are largely dependent on athlete self-report of playing activity and concussions, and estimates for the number of actual head impacts. Additionally, regular periods without head impacts during the football offseason may reduce the immediacy of head impact burden and its effect on concussion, unlike within-season analyses. Another important factor in the assessment of head impact burden may be the frequency of repetitive head impacts.^11^ The mechanism by which repetitive non-concussive head impacts contribute to the eventual onset of concussion likely involves disruption of the healing process within the brain, with earlier disruption having a more pronounced effect. Although this factor was indirectly included in this time-bound analysis, future laboratory-based studies are necessary to more clearly define the role of this important factor.

Trends from this study of head impact exposure by position were generally consistent with prior studies in demonstrating a greater number of recorded head impacts for the offensive and defensive linemen and the fewest number of recorded impacts for receivers and quarterbacks.^9^,^13^,^27^,^32^ Baugh *et al.* reported that offensive linemen participated in a greater number of full-contact practices than other playing positions,^2^ a trend that was generally consistent with the present results, wherein offensive linemen participated in the second highest number of preseason contact practices. Tight ends participated in the highest number of contact practices. The total number of head impacts across the preseason and regular season was also approximately consistent with prior studies using similar methods.^14^

### Limitations

A primary limitation of this analysis is that the present study was not able to track whether enrolled athletes were starters or back-ups. While the initial questionnaire included a question regarding starting status, that status was not able to be tracked and updated during the season. Starters would be expected to have more playing time during games and a higher number of repetitions during practice. Any differences in the percentage of starters versus back-ups between seasons could have contributed to the reported results regarding head impact burden in the preseason and regular season. Another possible limitation in this analysis is the accuracy of the Head Impact Telemetry System. In general, laboratory-based validation studies have reported reasonable accuracy for the HIT System in measuring the magnitude of head impacts,^4^,^30^ although other studies have reported higher error values for facemask impacts.^19^ However, the present focus on the number and not the magnitude of subconcussive head impacts may have mitigated the inaccuracy of the HIT System to some degree. Video verification of head impacts is a method that is often used to reduce the number of false positive and false negative impacts within a dataset and confirm the accuracy of the head impact measurement system. Given the extreme number of recorded head impacts in this study, occurring in practices, scrimmages, and games, video verification of head impacts was not possible. However, some steps were taken to reduce the number of false positive impacts. For example, the timing of recorded head impacts was compared to known team practice times and recorded events occurring outside of those time windows were eliminated. However, a lack of video verification of recorded head impacts should be acknowledged as a limitation of the current study. Justification for the present focus on the number of head impacts is derived from our prior study that indicated a stronger dependence of cumulative exposure (i.e., RWE) on the number and not severity of recorded head impacts.^34^

## Conclusions

In summary, this manuscript presents repetitive head impact exposure data for 342 NCAA Division I FBS college football athletes over the course of two seasons. This analysis has shown that weekly head impact burden was significantly greater during preseason training camp than the regular season, and preseason head impact exposure was significantly greater in 2017 compared to 2016. Given the likely association between repetitive head impact exposure and concussion, these data highlight a need to focus on reducing head impact exposure during preseason training camp. Despite rule changes intended to reduce exposure in the preseason by eliminating two-a-day practices, our data actually indicate an increase in total head impact burden during the preseason due primarily to increased contact intensity over the same number of practice sessions. Accordingly, results of this study highlight that tracking and reducing intensity of contact during practices may be more effective at reducing total head impact burden than reducing the number of contact sessions. Future efforts to reduce total exposure and concussion risk should focus more specifically on limiting contact activities or on improved training techniques to reduce the number of head impacts during practice throughout the season.
